# Vaccinia Virus Infection & Temporal Analysis of Virus Gene Expression: Part 2

**DOI:** 10.3791/1169

**Published:** 2009-04-10

**Authors:** Judy Yen, Ron Golan, Kathleen Rubins

**Affiliations:** Whitehead Institute for Biomedical Research, MIT - Massachusetts Institute of Technology

## Abstract

The family *Poxviridae* consists of large double-stranded DNA containing viruses that replicate exclusively in the cytoplasm of infected cells.  Members of the *orthopox* genus include variola, the causative agent of human small pox, monkeypox, and vaccinia (VAC), the prototypic member of the virus family.  Within the relatively large (~ 200 kb) vaccinia genome, three classes of genes are encoded: early, intermediate, and late.  While all three classes are transcribed by virally-encoded RNA polymerases, each class serves a different function in the life cycle of the virus.  Poxviruses utilize multiple strategies for modulation of the host cellular environment during infection. In order to understand regulation of both host and virus gene expression, we have utilized genome-wide approaches to analyze transcript abundance from both virus and host cells.  Here, we demonstrate time course infections of HeLa cells with Vaccinia virus and sampling RNA at several time points post-infection.  Both host and viral total RNA is isolated and amplified for hybridization to microarrays for analysis of gene expression.

**Figure:**
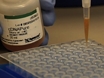


## Protocol

### Part 1: cDNA synthesis from RNA

1. Before using the Ambion Amino Allyl MessageAmp II kit, add the recommended volume of 100% ethanol to the wash buffers.
2. In PCR reaction tube, add between 100ng to 5µg of total RNA and 1µl of T7 oligo (dT) primer.  Bring the volume up to 12µl with nuclease-free water.
3. Incubate samples at 70°C for 10 min in a thermocycler.
4. Remove RNA samples from 70°C and centrifuge briefly.  Place on ice.
5. Prepare the 1^st^ Strand Synthesis master mix and keep at room temperature (with 10% overage for pipetting error). **(Table 1)**
6. Gently pipette Master Mix or flick to mix, and then centrifuge briefly.
7. Transfer 8µl of the Master Mix to each RNA sample.  Mix by pipetting up and down 3-4 times.
8. Incubate at 42°C for 2 hours in thermocycler.
9. Centrifuge samples briefly and place on ice.  Immediately proceed to the next step of dsDNA synthesis.
10. Prepare the 2^nd^ Strand Synthesis master mix on ice (with 10% overage for pipetting error). **(Table 2)**
11. Gently pipette Master Mix or flick to mix, and then centrifuge briefly.
12. Transfer 80µl to each sample and gently mix by pipetting up and down 3-4 times.
13. Incubate at 16°C for 2 hours in thermocycler.
14. After the 2 hour incubation, proceed with the cDNA clean-up step or freeze immediately at –20°C.

### Part 2: Double-stranded cDNA clean-up

1. Remove the cDNA Pure from the refrigerator and allow it to equilibrate to room temp for 30 minutes before use.  Shake the bottle to fully resuspend the magnetic cDNA binding beads before use.
2. Aliquot nuclease-free water into a 1.5mL tube and incubate at 50-60°C for at least 10 minutes during the previous 2hr incubation.
3. Add 180µl of cDNA Pure to each sample, and thoroughly mix by pipetting up and down.  Transfer the samples to a 96-well round-bottom plate.
4. Continue to mix the samples by gently shaking the plate on an orbital shaker for at least 2 minutes.
5. Move the plate to a magnetic stand to capture the magnetic beads.  Leave plate on the stand for approximately 6 minutes, or until the mixture becomes transparent and the binding beads have pelleted.
6. Carefully aspirate the supernatant with a vacuum aspirator without disturbing the magnetic beads.  Alternatively, carefully remove the supernatant with a pipette and discard the supernatant.
7. Remove the plate from the magnetic stand.
8. Add 150µl cDNA Wash Buffer to each well and shake the plate for 1 minute on the orbital shaker at moderate speed.  Beads will NOT disperse at this step, due to the low surface tension of the wash buffer.
9. Move the plate to a magnetic stand to capture the magnetic beads.
10. Carefully aspirate the supernatant with a vacuum aspirator without disturbing the magnetic beads.  Alternatively, carefully remove the supernatant with a pipette and discard the supernatant.
11. Remove the plate from the magnetic stand.
12. Repeat the wash a 2^nd^ time with 150µl cDNA Wash Buffer.
13. After the 2^nd^ wash, dry the beads by shaking the plate for 2 minutes on the orbital shaker at the maximum speed.  Do not overdry!
14. Elute the cDNA from the beads by adding 18µl of the preheated nuclease-free water to each sample.
15. Vigorously shake the plate for 3 minutes on the orbital shaker, then check to make sure the magnetic beads are fully dispersed.  If not, continue shaking.
16. Once the magnetic beads have fully dispersed, move the plate to a magnetic stand to capture the magnetic beads.
17. Carefully transfer the eluted cDNA (~16µl) to a new PCR plate (or PCR tubes).
18. Proceed directly to the next step, or freeze the cDNA at -20°C.

### Part 3: *in vitro* Transcription (IVT)

1. Prepare the IVT master mix at room temperature. **(Table 3)**
2. Gently pipette the Master Mix or flick to mix, and centrifuge briefly.
3. Add 24µl to each sample, and gently mix by pipetting up and down 3-4 times.
4. Incubate at 37°C for 14 hours in a thermocycler, then hold at 4°C until ready for the next step.

### Part 4: aRNA clean-up after IVT

1. Vortex the RNA binding beads briefly to obtain an even mixture before use.
2. Prepare the aRNA Binding Mix at room temperature. **(Table 4)** This can be done ahead of time--the prepared binding mix can be stored at room temperature for up to one week.
3. Mix well by vortexing.
4. Aliquot the aRNA Elution Buffer into a 1.5mL tube and incubate at 50-60°C for at least 10 minutes.
5. Add 70µl of the aRNA binding mix to each sample and mix well by pipetting up and down 3-4 times.
6. Transfer the samples from the PCR plate to a 96-well round-bottom plate.
7. Add 50µl of 100% isopropanol to each sample and mix well by pipetting up and down 3-4 times.
8. Gently shake the plate on an orbital shaker for at least 2 minutes to thoroughly mix the samples.
9. Move the plate to a magnetic stand to capture the magnetic beads.  Leave the plate on the stand until the mixture becomes transparent and the binding beads have pelleted.
10. Carefully aspirate the supernatant with a vacuum aspirator without disturbing the magnetic beads.  Alternatively, carefully remove the supernatant with a pipette and discard the supernatant.
11. Remove the plate from the magnetic stand.
12. Add 100µl aRNA Wash Solution to each well and shake the plate for 1 minute on the orbital shaker at moderate speed.  Beads may not fully disperse at this step.
13. Move the plate to a magnetic stand to capture the magnetic beads.
14. Carefully aspirate the supernatant with a vacuum aspirator without disturbing the magnetic beads.  Alternatively, carefully remove the supernatant with a pipette and discard the supernatant.
15. Remove the plate from the magnetic stand.
16. Repeat the wash a 2^nd^ time with 100µl aRNA Wash Solution.
17. After the 2^nd^ wash, dry the beads by shaking the plate for 1 minute on the orbital shaker at the maximum speed.  Do not overdry the samples!
18. Elute the aRNA from the beads by adding 40µl preheated aRNA Elution Buffer to each sample.
19. Vigorously shake the plate on the orbital shaker for 3 minutes, then check to make sure the magnetic beads are fully dispersed.  If not, continue shaking.
20. Once the magnetic beads have fully dispersed, move the plate to a magnetic stand to capture the magnetic beads.  The supernatant contains the cleaned-up amino-allyl incorporated aRNA.
21. Carefully transfer the eluted aRNA to a new PCR plate (or PCR tubes).
22. (Optional step) Check the RNA concentration of the samples by measuring 1.5µl on a NanoDrop spectrophotometer.
23. Immediately continue onto the next step, or store the aRNA at –80°C.

### Table 1: cDNA 1^st^ Strand Synthesis Master Mix

**Table:** 

**Reagent**	**Amount for 1 reaction**
10X First Strand Buffer	2 µl
dNTP Mix	4 µl
Ribonuclease Inhibitor	1 µl
Array Script	1 µl

### Table 2: cDNA 2^nd^ Strand Synthesis Master Mix

**Table:** 

**Reagent**	**Amount for 1 reaction**
Nuclease free water	63 µl
10X Second Strand Buffer	10 µl
dNTP Mix	4 µl
DNA Polymerase	2 µl
RNase H	1 µl

### Table 3: IVT Master Mix

**Table:** 

**Reagent**	**Amount for 1 reaction**
aaUTP solution (75 mM)	2 µl
ATP, CTP, GTP mix (25 mM)	12 µl
T7 UTP solution (75 mM)	2 µl
T7 10X Reaction Buffer	4 µl
T7 Enzyme Mix	4 µl

### Table 4: aRNA Binding Mix

**Table:** 

**Reagent**	**Amount for 1 reaction**
RNA Binding Beads*	10 µl
Bead Resuspension Solution*	4 µl
100% isopropanol**	6 µl
aRNA Binding Buffer Concentrate	50 µl

* Mix the RNA binding beads with the bead resuspension solution first.** Add the isopropanol and mix well before adding the aRNA binding buffer concentrate.

## Discussion

### Critical Steps

A second round of amplification is not advised as biases in array data have been observed.  Mixing carefully at each enzymatic step (1^st^ and 2^nd^ strand cDNA synthesis, IVT) is critical to obtaining good amplification yields, as is incubation of each enzymatic step at the appropriate temperature.  A PCR cycler with adjustable heated lid is preferred – even deviations as small as 2-3 degrees during IVT in an air hybridization oven or water bath hybridization can significantly affect yield of amplified product.

### Application/Significance

The labeled RNA resulting from this protocol can be hybridized to human, viral, or custom microarrays to assess gene expression responses to infected cells in culture.  Microarray platforms vary, so follow manufacturer instructions for preparation of hybridization mixture from labeled probe.

Using a custom designed poxvirus array^1^, we were able to classify genes into the general categories of “early” or “late” based on timing of hybridization signal and whether or not viral DNA replication was required for transcript detection.  We observed the expected functional categories of genes in each temporal class (i.e., expected early, intermediate and late genes) variation as to the exact timing of transcription.

The methods utilized in this work are able to predict virus genes transcribed early or late in the replication cycle, but have more difficulty distinguishing early-only versus genes with an early and late promoter since transcripts with a dual early/late promoter may persist and be detected at late times. In addition, run-through transcription of late viral genes may affect signal at a given probe/spot on the array, as the RNA hybridizing to the array may have come from the designated ORF or an upstream ORF.  Tiling arrays have attempted to resolve this issue, however challenges remain in detecting run through transcription using hybridization based approaches^2,3,4^.

Host transcriptional patterns can also be assessed using these methods.  However, vaccinia encodes a variety of mechanisms to inhibit host responses, and host transcriptional responses may be diminished compared to other stimuli^5,6,7,8^.  Since the expression of many genes involved in host defense is altered after infection, the contribution of viral genes that counteract host immune responses should therefore be taken into consideration.

Utilizing these methods, a map of the transcriptional timing of all viral genes can be identified and used to interrogate functions of unknown viral genes.  In addition, these methods can be utilized to dissect the intricate dialogue between virus and host.  These methods are broadly applicable to other host-pathogen infection systems.  If the pathogen of interest does not have polyadenylated mRNAs, alternative methods can be used to directly label the total RNA, without linear amplification.  By analyzing both host and virus gene expression during synchronous infection, these methods allow us to gain insight into virus interaction with the host cellular environment as well as host counter-defenses against virus infection.
